# Motor Imagery Training With Neurofeedback From the Frontal Pole Facilitated Sensorimotor Cortical Activity and Improved Hand Dexterity

**DOI:** 10.3389/fnins.2020.00034

**Published:** 2020-01-29

**Authors:** Yuya Ota, Kouichi Takamoto, Susumu Urakawa, Hiroshi Nishimaru, Jumpei Matsumoto, Yusaku Takamura, Masahito Mihara, Taketoshi Ono, Hisao Nishijo

**Affiliations:** ^1^System Emotional Science, Faculty of Medicine, University of Toyama, Toyama, Japan; ^2^Department of Sports and Health Sciences, Faculty of Human Sciences, University of East Asia, Shimonoseki, Japan; ^3^Department of Musculoskeletal Functional Research and Regeneration, Graduate School of Biomedical and Health Sciences, Hiroshima University, Hiroshima, Japan; ^4^Department of Neurology, Kawasaki Medical School, Okayama, Japan

**Keywords:** fNIRS, neurofeedback, frontal pole, motor rehabilitation, primary motor cortex

## Abstract

To develop a real-time neurofeedback system from the anterior prefrontal cortex (aPFC) using functional near-infrared spectroscopy (fNIRS) for motor rehabilitation, we investigated the effects of motor imagery training with neurofeedback from the aPFC on hand dexterity and cerebral hemodynamic activity during a motor rehabilitation task. Thirty-one right-handed healthy subjects participated in this study. They received motor imagery training six times for 2 weeks under fNIRS neurofeedback from the aPFC, in which they were instructed to increase aPFC activity. The real group subjects (*n* = 16) were shown real fNIRS neurofeedback signals from the aPFC, whereas the sham group subjects (*n* = 15) were shown irrelevant randomized signals during neurofeedback training. Before and after the training, hand dexterity was assessed by a motor rehabilitation task, during which cerebral hemodynamic activity was also measured. The results indicated that aPFC activity was increased during the training, and performance improvement rates in the rehabilitation task after the training was increased in the real group when compared with the sham group. Improvement rates of mean aPFC activity across the training were positively correlated with performance improvement rates in the motor rehabilitation task. During the motor rehabilitation task after the training, the hemodynamic activity in the left somatosensory motor-related areas [premotor area (PM), primary motor area (M1), and primary somatosensory area (S1)] was increased in the real group, whereas the hemodynamic activity was increased in the supplementary motor area in the sham group. This hemodynamic activity increases in the somatosensory motor-related areas after the training correlated with aPFC activity during the last 2 days of motor imagery training. Furthermore, improvement rates of M1 hemodynamic activity after the training was positively correlated with performance improvement rates in the motor rehabilitation task. The results suggest that the aPFC might shape activity in the somatosensory motor-related areas to improve hand dexterity. These findings further suggest that the motor imagery training using neurofeedback signals from the aPFC might be useful to patients with motor disability.

## Introduction

Motor rehabilitation is fundamental to management of patients with stroke as well as chronic neurological disorders such as Parkinson’s disease, Alzheimer’s disease, vestibular disease, etc. (Johansson, 2011; Cheng et al., 2012; Hatem et al., 2016). These neurological disorders increased in the last 25 years, and the number of patients in need of neurological cares will increase in the next decades (GBD 2015 Neurological Disorders Collaborator Group, 2015). Hemiparesis of the upper limb is the most common motor disturbance after a stroke. It affects more than 80% of patients in an acute phase, and more than 40% in a chronic phase (Cramer et al., 1997). In Parkinson’s disease, the reduction of fine hand skills seriously affects daily activities (Raggi et al., 2011). In mild cognitive impairment and Alzheimer’s disease, fine motor function such as hand dexterity is disturbed, impairing activities of daily living (ADL) (Scherder et al., 2008; de Paula et al., 2016). These findings suggest that to increase the patients’ quality of life (QOL), and also to reduce medical costs, appropriate rehabilitation methods for upper limbs should be developed.

Motor rehabilitation ability is associated with motor skill learning (Hanlon, 1996). Motor skill learning and the resultant formation of motor memories can be defined as an improvement of motor skills through practice (Brem et al., 2013). Repetitive performance of a rehabilitation task effectively improves motor skills of the upper extremity, which is attributed to motor skill learning based on changes in brain neural circuits, especially on those in the primary motor cortex (M1) (Hatakenaka et al., 2007; Papale and Hooks, 2018). Neurofeedback is biofeedback in which sensory (usually visual or auditory) signals reflecting real-time neural activity are displayed to subjects so that they can learn to modulate activity in targeted neural substrates involved in specific behaviors or brain functions (Sitaram et al., 2016; Wang et al., 2018). Neurofeedback could induce specific neural activation patterns in target brain areas (Shibata et al., 2011; Sitaram et al., 2016), suggesting that neurofeedback training could induce changes in neural circuits for motor skill learning and, consequently, could be used for motor rehabilitation training. It is also noted that neurofeedback training could be beneficial to patients with motor disability such as stroke since patients do not need to make overt behaviors during training. Also, neurofeedback has been recently applied to motor rehabilitation in stroke patients as well as healthy adults (Mihara et al., 2012, 2013; Fujimoto et al., 2017; Wang et al., 2018). These previous studies targeted the sensorimotor related areas as neurofeedback sources: M1, primary somatosensory cortex (S1), premotor cortex (PM), and supplementary motor area (SMA).

We previously reported that hemodynamic activity in the anterior part of the prefrontal cortex (aPFC), which corresponds to the frontal pole (Brodmann area 10), was increased during motor learning in a motor rehabilitation task of hand dexterity, and correlated with the performance improvement rate in healthy subjects (Ishikuro et al., 2014). Furthermore, anodal stimulation of the aPFC improved hand dexterity in the same motor rehabilitation task in both healthy adults and patients with Parkinson’s disease (Ishikuro et al., 2014, 2018). These previous results suggest that the aPFC facilitates motor skill learning, and further suggest that training with neurofeedback from the aPFC might be useful for motor rehabilitation of the hand. To develop a neurofeedback system, we applied functional near-infrared spectroscopy (fNIRS) to measure aPFC activity as neurofeedback signals. fNIRS is a neuroimaging technique that can detect changes in oxygenated-hemoglobin (Oxy-Hb), deoxygenated-hemoglobin (Deoxy-Hb), and total hemoglobin (Total-Hb) in the cerebral cortex associated with local cortical activity based on neurovascular coupling (Ferrari and Quaresima, 2012; Quaresima and Ferrari, 2019). fNIRS can be used with less body and head restraint in relatively larger spaces. Thus, fNIRS allows us to measure brain activity under conditions similar to actual clinical environments when compared with the other imaging methods such as functional magnetic resonance imaging (fMRI) and positron emission tomography (PET).

In this study, we hypothesized that neurofeedback training targeting the aPFC would improve hand dexterity through its effects on the sensorimotor cortex. To investigate the effects of training with neurofeedback from the aPFC on hand motor dexterity and cortical hemodynamic activity, we analyzed changes in hand dexterity and cortical hemodynamic activity during a motor rehabilitation task for hand dexterity before and after neurofeedback training. Here we report that cerebral hemodynamic activity in the somatosensory motor-related areas was increased during the motor rehabilitation task after neurofeedback training, which correlated to the aPFC activity during training. Furthermore, improvement rates of M1 hemodynamic activity after the training was associated with performance improvement rates in the motor rehabilitation task.

## Materials and Methods

### Subjects

The inclusion criterion was right-handed healthy adults who had no history of neurological and psychological disorders, and no experience of neurofeedback training (Dieterich et al., 2003). Histories of neurological and psychological disorders were assessed based on the subjects’ self-reports. Handedness was determined by the Edinburgh Handedness Inventory (Oldfield, 1971), and all subjects included were right-handed. A total of 31 subjects participated in the current study [25.4 ± 0.7, mean age ± standard error (SE), ranging from 20 to 33 years old; 17 males and 14 females]. The subjects were randomly grouped into two groups: real group subjects (*n* = 16; nine males and seven females) were shown real fNIRS neurofeedback signals from the aPFC, while sham group subjects (*n* = 15; eight males and seven females) were shown irrelevant randomized signals during neurofeedback training. The subjects were blinded to subject grouping. All subjects were treated in strict compliance with the Declaration of Helsinki and the United States Code of Federal Regulations for the protection of human participants. We obtained written informed consents from all subjects prior to experiments. The present experimental protocol was approved by the Ethical Committee of Human Experiments at the University of Toyama.

### Sample Size

The sample size for the comparison of two independent samples (two-tailed *t*-test) was estimated using G^∗^Power, a tool to compute statistical power analyses^1^ (Faul et al., 2007). Data in the previous study (Ishikuro et al., 2014), in which cortical hemodynamics and peg task performance were analyzed, were used for this sample size estimation. The analysis indicated an *n* = 11 for each group based on the following conditions; level of significance = 0.05, statistical power = 0.95, mean and standard deviation (SD) in group 1 = 11.4 and 1.31, respectively, and mean and SD in group 2 = 9.0 and 1.55, respectively.

### Experimental Procedures

**FIGURE 1:**
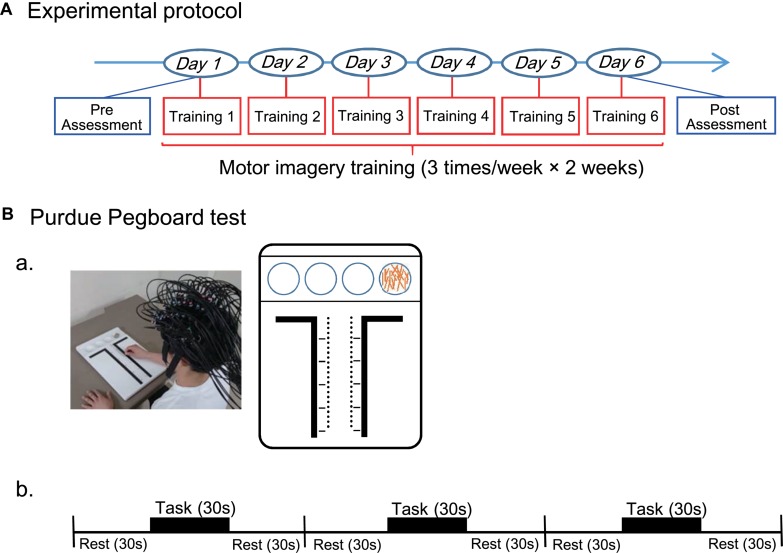
Experimental protocol throughout this study **(A)** and that for the Purdue Pegboard test **(B)**. **(A)** The entire experimental protocol. The experimental protocol composed of three sessions; pre (before training)-assessment session, motor imagery training session, and post (after training)-assessment session. In the motor imagery training session, the subjects received motor imagery training three times a week for 2 weeks. Hand dexterity was assessed using the Purdue Pegboard test in the pre- and post-assessment sessions. **(B)** The Purdue Pegboard test protocol. **(a)** A photo of a subject with an fNIRS head cap during Purdue Pegboard testing and schematic illustration of the Purdue Pegboard are shown. The Purdue Pegboard has four cups in the upper side and two rows of 25 holes each arranged vertically in the center of the board. **(b)** The Purdue Pegboard test consisted of three blocks, and each block had three phases: each phase for 30 s (rest, task, and rest).

Subjects were randomly grouped into two groups; (1) A real group (*n* = 16) that was shown real fNIRS neurofeedback signals, and (2) A sham group (*n* = 15) that was shown irrelevant randomized signals during neurofeedback training. The experimental protocol was composed of three sessions; pre (before training)-assessment session, motor imagery training session, and post (after training)-assessment session (Figure 1A). In the pre-assessment session, after an fNIRS head cap and probes have been set on the head (see below in detail), hand dexterity was assessed by using the Purdue Pegboard test (see below in detail) as the baseline status. In the motor imagery training session, the subjects received motor imagery training three times a week for 2 weeks. After the last session of the training, hand dexterity was reassessed using the same Purdue Pegboard test in the post-assessment session. In the Purdue Pegboard test in the pre- and post-assessment sessions, whole-brain hemodynamic activity was also recorded (see below in detail). In the motor imagery training sessions, cortical activation in the anterior prefrontal cortex (aPFC: Brodmann area 10) (Ramnani and Owen, 2004) was assessed by real-time analysis of fNIRS signals from the aPFC (see below in detail).

### The Purdue Pegboard Test

The Purdue Pegboard test (Mathiowetz et al., 1986; Vasylenko et al., 2018) was used to evaluate hand dexterity before and after the training session. Subjects sat in a chair in front of a table 755 mm in height. The Purdue Pegboard (Model 32020A, Lafayette Instrument, Co. Ltd., IN, United States) was placed on the table (Figure 1Ba). The Purdue Pegboard had four cups in the upper side and two rows of 25 holes each arranged vertically in the center of the board. The 25 pins (pegs) were initially placed in the extreme right cup. In the pre-assessment, the subjects received brief instructions from the experimenter. After the instructions, the subjects were allowed to briefly perform the task for practice.

In the Purdue Pegboard test, the subjects picked up one of the pegs from the right-handed cup and put into a hole using their right hands, starting at the top of the right row to the bottom. The subjects were asked to put as many pegs as possible into the holes within a 30-s period in each block of the task. The test consisted of three blocks of three phases: rest, task, and rest (each phase for 30 s) (Figure 1Bb). Thus, the actual inter-task rest period was 60 s [a last resting period (30 s) in the previous block plus an initial resting period (30 s) in the next block]. Performance in the Purdue Pegboard test was assessed by counting the number of pegs put into holes.

After the pre-assessment of the Purdue Pegboard test, motor imagery ability of the subjects was assessed using Movement Imagery Questionnaire-Revised Japanese Version (JMIQ-R) (Hasagawa and Hoshino, 2002). The subjects were required to score their motor imageries of four actions using own extremities.

### Motor Imagery Training

**FIGURE 2:**
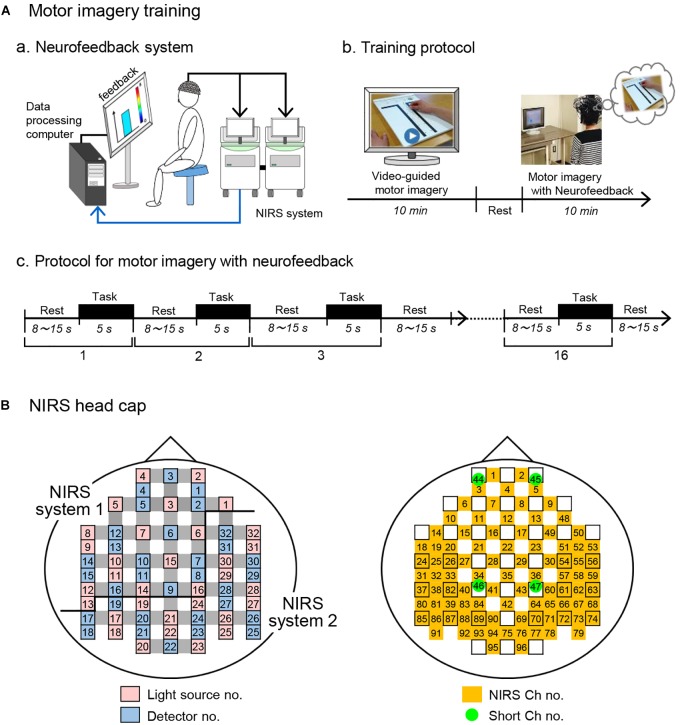
Motor imagery training using fNIRS neurofeedback system. **(A)** Motor imagery training. **(a)** A schematic figure of the fNIRS-mediated neurofeedback system. **(b)** Protocol for motor imagery training. Motor imagery training consisted of video-guided motor imagery and motor imagery with neurofeedback. **(c)** Protocol for motor imagery with neurofeedback. The training protocol consisted of 16 trials. Each trial consists of 5-s motor imagery and 8 -15-s rest periods. **(B)** Arrangement of probes and channels in an fNIRS head cap. As the source of feedback signals, 5 fNIRS channels in the aPFC (Ch 1-5) were used. NIRS ch no., NIRS channel no. with probe distance of 3.0 cm; Short ch no., with probe distance of 1.5 cm.

After an fNIRS head cap and optodes were set on the head, the subjects sat in a chair in front of a screen and were asked to open their eyes to look at the screen (Figure 2Aa). The motor imagery training composed of (Figure 2Ab): (1) video-guided motor imagery without neurofeedback for 10 min, and (2) motor imagery with neurofeedback for 10 min (Mihara et al., 2013). In the video-guided motor imagery, the subjects in both groups were asked to perform motor imagery in the Purdue Pegboard test: picking up one of the pegs from a cup and putting it into a hole with the right hand following video instructions. After a short break of 1–2 min, motor imagery training with neurofeedback was started. In this second training, a bar to go up and down as real or randomized fNIRS feedback signals from the aPFC was shown to the subjects. The subjects in both groups were asked to look at the feedback bar as neurofeedback from the aPFC on the screen and to perform motor imagery of the Purdue Pegboard test (to keep the height and color of the feedback bar at the elevated levels; see below for details). The sham group was shown the feedback bar, height and color of which did not reflect real fNIRS signals.

Each training of motor imagery with neurofeedback consisted of 16 trials consisting of a 5-s period of the motor imagery task followed by inter-task rest periods ranging from 8 to 15 s (Figure 2Ac). To prevent prediction of task start by the subjects, the inter-task rest period was pseudorandomly set (mean resting time, 11.19 ± 0.53 s). In response to beep sounds indicating the start of each trial, the subjects were asked to perform the motor imagery as if they actually moved their right fingers and hand in the Purdue Pegboard test. Throughout the motor imagery training, behaviors of each subject were recorded in a video camera (HC-V480M, Panasonic, Co. Ltd., Osaka, Japan) to observe their posture, eyes, hand, and finger movements. All subjects opened their eyes and looked at the screen without overt changes of their posture, and overt hand and finger movements were not observed (data not shown).

### Measurements of Hemodynamic Activity Using fNIRS

Two fNIRS systems (OMM 3000, Shimadzu, Co. Ltd., Kyoto, Japan) were used to measure changes in brain hemodynamic activity from the bilateral hemispheres. To measure data as the integrated system, the two fNIRS systems were connected with Ethernet and SYNC cables. One fNIRS system was used as the master, the clock signal was synchronized using the SYNC cable, and the measurement control commands were synchronized by the TCP/IP protocol using Ethernet cable. The systems were automatically calibrated using target measurement condition in advance before the experiment so that all NIRS signals were comparable.

An fNIRS head cap was placed on the subject’s head. The optodes for the fNIRS instruments were fixed on the head cap and the bottom horizontal line of the frontal optodes was placed according to the international 10–20 EEG system (2 cm posterior to the subject’s Fpz in the current study) (Takeuchi et al., 2009; Takamoto et al., 2010; Takakura et al., 2015). The fNIRS systems used three different wavelengths (780, 805, and 830 nm) to detect hemodynamics (oxygenated Hb [Oxy-Hb], deoxygenated Hb [Deoxy-Hb] and Total-Hb [Oxy-Hb + Deoxy-Hb]), which were estimated using a modified Lambert–Beer law (Seiyama et al., 1988; Wray et al., 1988).

The light detector optode detected hemodynamic signals around the midpoints (called “channels”) between the light source and detector optodes. The hemodynamic signals include different information depending on the optode distance between light sources and detectors (Fukui et al., 2003; Niederer et al., 2008; Ishikuro et al., 2014). The fNIRS signals from optodes with 3 cm include both cerebral (brain) and extra-cerebral (scalp, skull, and cerebrospinal fluid) components, and the signals from optodes with 1.5 cm reflect extra-cerebral components. In this study, multi-distance optode arrangement was applied to remove artifacts and extract cerebral hemodynamics from all hemodynamic responses that included both extra-cerebral and cerebral components (Schytz et al., 2009; Nakamichi et al., 2018). Furthermore, to record from the somatosensory motor-related areas more densely than a conventional optode arrangement in a channel lattice of 30 × 30 mm, extra sources and probes were placed in the bilateral somatosensory motor-related areas so that fNIRS signals could be recorded in a channel lattice of 15 × 15 mm (e.g., extra-source No. 9, 11, 13, 19, 18, 22; extra-detector No. 13, 15, 19, 18, 21 in the left hemisphere; Figure 2B). This high density optode arrangement is reported to improve spatial resolution (Yamamoto et al., 2002; White and Culver, 2010). Thus, hemodynamic signals were measured from 92 channels at 4 Hz using 32 light-source probes and 28 light-detector probes (Figure 2B), and the optodes were placed across from each other at 3 cm by an adjustment mechanism based on the Guss-Bonnet theorem (Banados et al., 1994; Cummings, 2001). Another 4 detectors (No. 1, 4, 8, 11) were positioned 1.5 cm from source optodes, resulting in 4 channels (Ch 44-47) with short distances (1.5 cm). The short channels were placed in the bilateral aPFC and somatosensory motor-related areas. The positions of the short channels were determined, so that distance between the long (3.0 cm) and short (1.5 cm) channels was relatively similar across the head.

After recording, 3-dimensional locations of the optodes were measured using a Digitizer (FASTRAK, Polhemus Inc., United States) with reference to the vertex (Cz), nasion and bilateral external auditory meatus. The anatomical locations of the fNIRS optodes and channels in each subject were normalized to standard coordinates in the Montreal Neurological Institute (MNI) coordinate system (Singh et al., 2005). Furthermore, we identified the cortical regions covered by each channel using the MRIcro software^2^, as well as the Brodmann’s area image and automated anatomic labeling image (Tzourio-Mazoyer et al., 2002).

### fNIRS-Mediated Neurofeedback System

In this study, we analyzed Oxy-Hb signals as cortical activity. Previous studies reported that Oxy-Hb was correlated with fMRI BOLD signals and it might be the most consistent parameter for cortical activity (Hoshi et al., 2001; Strangman et al., 2002; Yamamoto and Kato, 2002). fNIRS signals were sampled at 4 Hz, and the data were transferred to the neurofeedback system online (Figure 2Aa). As the source of feedbacks from a region of interest (ROI), 5 fNIRS channels (Ch 1-5 in Figure 2B) in the aPFC were used. Transferred fNIRS signals were initially filtered with a lowcut filter (0.0125 Hz). A sliding-windows general linear model (GLM) analysis with a least-square estimation was used for real-time analysis of signal changes by in-house programs in MATLAB (R2014b; Math Works, Natick, MA, United States). The detailed methods for real-time analysis of fNIRS signals have been reported previously (Mihara et al., 2012). The window included 80 data points, which covered at least one cycle of the task (5 s) and rest (8–15 s) periods. Each window was measured for 20 s at 4 Hz. To eliminate contamination of the extra-cerebral components, such as the influence of scalp blood flow, respiration, heart rate, and motion artifacts, a principal component analysis was simultaneously performed using data from the short distance channels (Ch 44 and 45 in Figure 2B). The primary principal component was included in the model as a regressor. The *t-*values were used to estimate changes in cortical activation.

The maximal *t*-values from the feedback ROI were used as the cortical feedback signals. The height and color of the feedback bar varied from 0 (blue) to 8 (red), according to the *t*-value. The *t*-values > 2.0 indicated significant activation (approximately *P* < 0.05). If *t*-values from all the 5 fNIRS channels were lower than zero, suggesting no significant cortical activation, the feedback bar was set to zero. In the sham group, random signals regardless of their own aPFC activation were generated as neurofeedback signals from prerecorded data of aPFC activity from other individuals. The prerecorded data were randomly selected from pooled data that were recorded during the same task in the real feedback condition. Thus, the height and color of the feedback bar on the screen reflected the real-time fNIRS signals in the real group, but the randomly selected prerecorded data in the sham group. The subjects in both groups were asked to keep the height and color of the feedback bar at higher levels (Mihara et al., 2013; Fujimoto et al., 2017).

### Data Analysis

#### Behavioral Measures

To evaluate the hand dexterity, peg scores, defined as the number of the pegs put into the holes, were estimated on each assessment before and after the motor imagery training, and the scores from three blocks were averaged. “Performance gain” was defined as the peg score in the post-assessment divided by that in the pre-assessment in individual subjects. Then, the mean performance gain of the two groups was compared using unpaired *t*-test.

#### fNIRS Data in the Feedback ROI During Motor Imagery Training

To compare progress in motor imagery training between the two groups, feedback signals (Oxy-Hb) were analyzed. Early training data (trials 1–4 in Figure 2Ac) from each training day were removed from the analysis due to data instability (Fujimoto et al., 2017). Then, the mean *t*-values of the 5 channels (Ch 1-5 in Figure 2B) in trials 5–16 were averaged for each subject in each training day, and the mean *t*-values for each day in each group were estimated. Finally, the averaged mean *t*-values across the 6 training days were compared between the two groups using paired *t*-test. To evaluate the effects of neurofeedback training on performance gain, the relationships between performance gain and improvement rate of hemodynamic activity (Oxy-Hb gain) in the feedback ROI during training were analyzed by simple regression analysis. The Oxy-Hb gain in the feedback ROI was defined as averaged *t*-values across Ch 1-5 on training day 6 divided by those on training day 1 in individual subjects.

We also analyzed temporal changes of cerebral hemodynamic responses (Oxy-Hb, Deoxy-Hb, and Total-Hb) during motor imagery training in the aPFC. First, cerebral hemodynamic responses were estimated by simple-subtraction methods (Schytz et al., 2009; Nakamichi et al., 2018): [the whole signals with probe distance of 3.0 cm in the aPFC] minus [the extra-cerebral signals with probe distance of 1.5 cm, located nearest to corresponding whole signals]. The subtracted cerebral signals were filtered with a bandpass filter (0.01–0.1 Hz) to reduce long-term baseline drift and autonomic responses such as cardiac or respiratory activity (Tong et al., 2011; Yasumura et al., 2014). The fNIRS signals were then summed and averaged across the 12 trials (from 5th to 16th trials except the early 4 trials) in each training day. The summed data were corrected for baseline activity from −3 to 0 s before the start of motor imagery (start beep tone).

#### fNIRS Data During Assessment of Hand Dexterity (Purdue Pegboard Test)

The cerebral hemodynamic activity (Oxy-Hb, Deoxy-Hb, and Total-Hb) was similarly computed using simple-subtraction methods (see above). In this study, we analyzed Oxy-Hb signals as cortical activity (see above). Oxy-Hb signals were analyzed using a mass univariate GLM by statistical parametric mapping on NIRS-SPM software^3^ (version 4.1) (Ye et al., 2009). In the group analysis, SPM *t*-statistic maps on the standardized brain in the MNI coordinate system were generated. Statistical significant level was set at an uncorrected threshold of *P* < 0.001 (Thornton et al., 2011; Kim et al., 2017) and the threshold setting of cluster-extent was 50 sequence (Woo et al., 2014; Theisen et al., 2017; Bansal and Peterson, 2018).

The above group analysis indicated activation in the several cortical areas including the left, but not right, somatosensory motor-related areas in the real group and SMA in the sham groups (see section “Results”). We investigated the relationship between hand dexterity during Purdue Pegboard testing and hemodynamic cortical activity in these activated areas using simple regression analysis. First, five cortical regions that showed significant activation in the SPM t-statistic maps in the post-assessment were selected as ROIs; the left premotor area (L-PM), left primary motor area (L-M1) for the hand (lateral L-M1), L-M1 except lateral L-M1 (medial L-M1), left primary somatosensory area (L-S1), and SMA. The L-M1 was divided into two parts based on the X coordinate of the MNI coordinates: the hand motor area (lateral L-M1: area with X ≤ −30) and the remaining area (medial L-M1: area with X ≥ −29) (Stoeckel et al., 2009; Hadoush et al., 2011; Lapborisuth et al., 2017; Schellekens et al., 2018). Second, in each ROI, the averaged *t-*values were calculated in the pre- and post-assessment sessions. Then, Oxy-Hb gain was calculated in each ROI as mean *t-*value in the post-assessment divided by that in the pre-assessment. Finally, in each ROI, a simple regression analysis was performed to analyze relationships between Oxy-Hb gain and performance gain. In these regression analyses, outliers were detected by residual analysis and were removed before the analyses. Data with standardized residuals larger than 3.29 were defined as outliers (Cook and Weisberg, 1982).

To investigate the effects of neurofeedback training on cortical activation during the Purdue Pegboard test, the relationships between the mean *t*-values in the feedback ROI on each training day and the mean *t*-values in the activated areas during the Purdue Pegboard test in the post-assessment were analyzed using simple regression analysis.

### Statistical Analysis

Data normality was assessed by the Shapiro–Wilk test. Homogeneity of variance was assessed by Levene’s test. Data between the real and sham groups were compared using Student’s *t*-test (or Mann–Whitney *U*-test) and analysis of variance (ANOVA). The simple regression analysis was used to investigate data correlation using the data in the all subjects as well as those in the real group. These statistical analyses were performed using SPSS statistical package version 19.0 (IBM, Co. Ltd., New York, NY, United States). The statistical significant level was set at *P* < 0.05.

## Results

### Baseline Characteristics

The baseline characteristics of the two groups are shown in Table 1. The mean (±SE) age in the real group was 25.4 ± 0.9 years, and that in the sham group was 25.3 ± 1.1 years. There were no significant differences between the two groups in terms of handedness [Student’s *t*-test; *T*(29) = 0.746, *P* > 0.05], age [Student’s *t*-test; *T*(29) = 0.029, *P* > 0.05], and sex [chi-squared test; χ^2^(1) = 0.027, *P* > 0.05].

**TABLE 1:** Baseline subject characteristics.

	**Real group**	**Sham group**
	**(*N* = 16)**	**(*N* = 15)**
Male (*N*)	9	8
Female (*N*)	7	7
Age (years)	25.4 ± 0.9	25.3 ± 1.1
Handedness (%)	89.2 ± 2.8	85.2 ± 4.7

*Age (years) and Handedness (%) are presented as mean ± SE. There were no significant differences between the two groups in terms of sex (Chi-squared test, *P* > 0.05), age and handedness (unpaired *t*-test, *P* > 0.05).*

### Performance in the Purdue Pegboard Test

**FIGURE 3:**
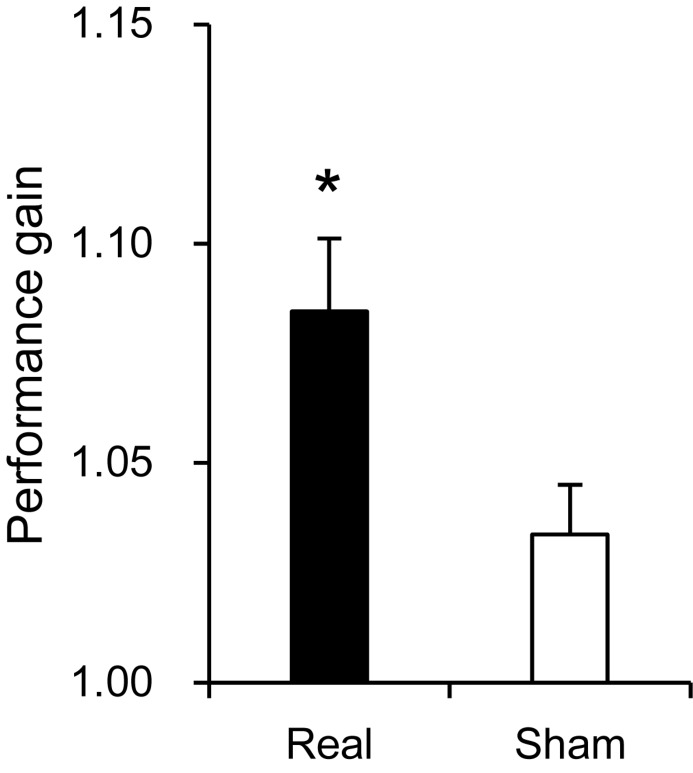
Comparison of performance gain between the real and sham groups. *P < 0.05.

Hand dexterity was assessed by using the Purdue Pegboard test. Peg scores in the pre-assessment session were used as a control before training, and there was no significant difference in scores between the two groups (14.67 ± 0.367 in the real group, 15.76 ± 0.475 in the sham group) [Mann–Whitney *U*-test; *U*(16, 15) = 71.00, *P* > 0.05]. These mean scores are comparable to those of the normative data (around 15–16) of the healthy adults in their twenties and thirties (Yeudall et al., 1986). However, performance gain was significantly higher in the real group than the sham group (1.085 ± 0.017 in the real group, 1.034 ± 0.011 in the sham group) [Student’s *t*-test; *T*(29) = 2.427, *P* < 0.05] (Figure 3).

After the pre-assessment of the Purdue Pegboard test, motor imagery ability of the subjects was assessed using the JMIQ-R. The mean total score in the real group was 42.94 ± 2.21, and that in the sham group was 43.93 ± 2.35. There was no significant difference in total sores between the real and sham groups [Student’s *t*-test; *T*(29) = 0.299, *P* > 0.05].

### Effects of Motor Imagery Training on aPFC Activity and Performance Gain

**FIGURE 4:**
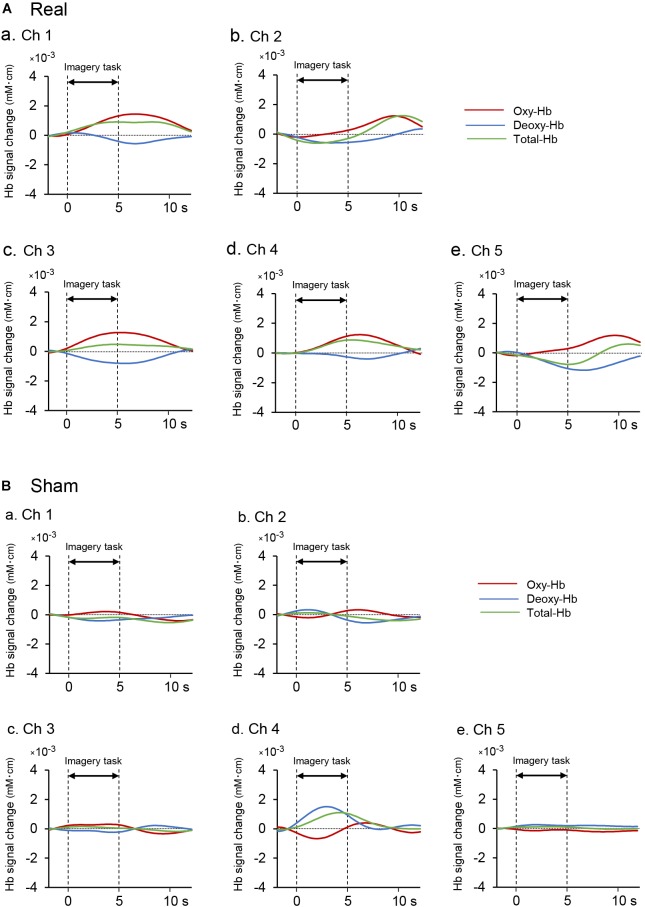
Examples of hemodynamic responses in the feedback ROI (Ch 1-5) during motor imagery training in the real **(Aa–Ae)** and sham **(Ba-Be)** groups. **(A)** Hemodynamic responses in one subject of the real group. In Ch 1, 3, and 4, Oxy-Hb and Total-Hb concentrations increased after task onset, whereas Deoxy-Hb concentration gradually decreased during neurofeedback training. **(B)** Hemodynamic responses in one subject of the sham group. There were no apparent increases in Oxy-Hb concentration after the task onset.

Figure 4A shows examples of cerebral hemodynamic responses in the 5 fNIRS channels in the aPFC during motor imagery training on day 6 in one subject of the real group. In Ch 1, 3, and 4, Oxy-Hb and Total-Hb concentration increased after onset, whereas Deoxy-Hb concentration gradually decreased during the task period. In Ch 2 and 5, Oxy-Hb concentration slightly increased during the task period, whereas Deoxy-Hb and Total-Hb concentrations gradually decreased after the onset. Figure 4B shows examples of the comparable data in one subject of the sham group. There were no apparent increases in Oxy-Hb concentration after the task onset.

**FIGURE 5:**
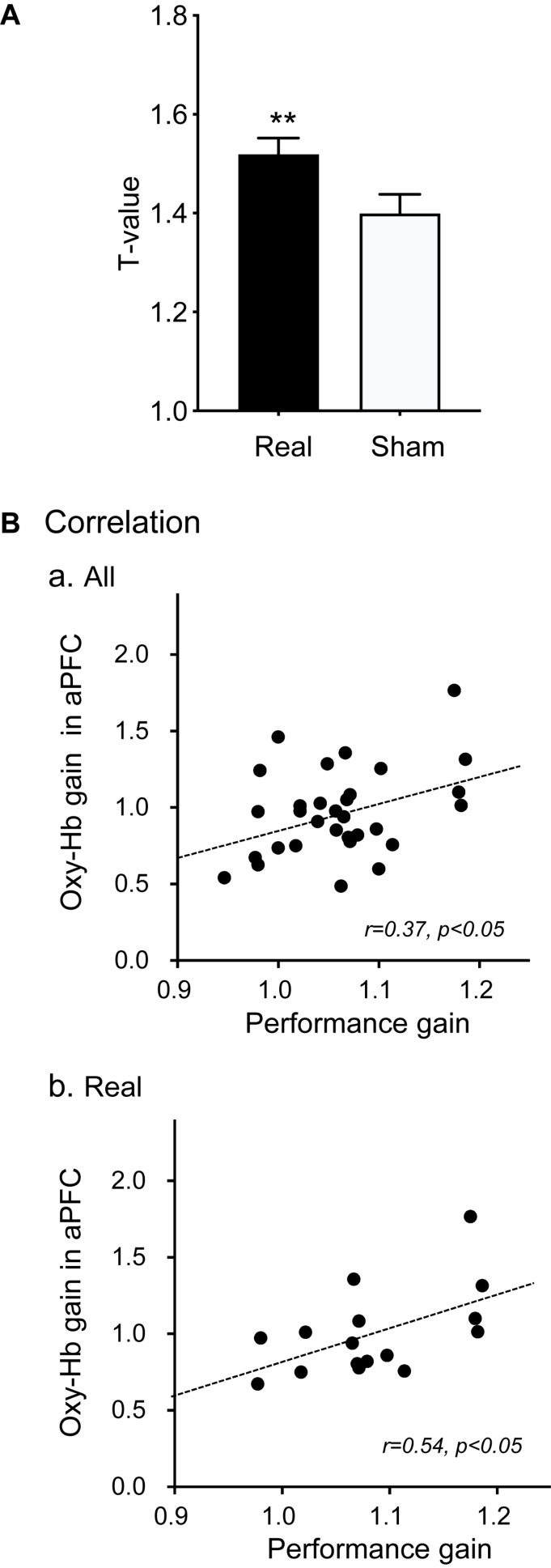
Effects of motor imagery training on aPFC activity **(A)** and performance gain **(B)**. **(A)** Comparison of cortical activation (mean *t*-values) in the aPFC during the motor imagery task between the real and sham groups. **P < 0.01. **(B)** Correlation between Oxy-Hb gain in the aPFC in motor imagery training and hand performance gain in the Purdue Pegboard test in all subjects **(a)** and in the real group **(b)**.

Figure 5A shows a comparison of the mean *t*-values in the 5 channels of the aPFC across the 6 training days between the real and sham groups. The results showed that the mean *t*-values were significantly higher in the real group when compared with the sham group (1.520 ± 0.032 in the real group, 1.400 ± 0.039 in the sham group) [paired *t*-test; *T*(5) = 4.383, *P* < 0.01]. Trends of changes in mean *t*-values in the aPFC during motor imagery training over the trials in each training day are shown in Supplementary Figure 1. The difference between the two groups in Figure 5A could be ascribed to the difference in the appearance of the presented bar between the two groups. However, there was no significant difference in the height of the feedback bar on the screen between the real and sham feedback groups (2.274 ± 0.165 in the real group, 2.270 ± 0.274 in the sham group) [Mann–Whitney *U*-test; *U*(16, 15) = 84.00, *P* > 0.05]. This indicated that the observed significant difference in the mean *t*-values of the aPFC signals between the two groups was not due to the difference in the screen bar.

To analyze the effects of neurofeedback training on hand dexterity, the relationships between performance gain in the Purdue Pegboard test and Oxy-Hb gain in the feedback ROI (aPFC) during motor imagery training were analyzed (Figure 5B). When the data of all subjects were analyzed (Figure 5Ba), the Oxy-Hb gain in the feedback ROI was significantly and positively correlated with performance gain [*r* = 0.37, *F*(1,29) = 4.726, *P* < 0.05]. When the data were confined to the real group (Figure 5Bb), there was also a significant positive correlation between the Oxy-Hb gain in the aPFC and the performance gain [*r* = 0.54, *F*(1,14) = 5.841, *P* < 0.05].

### Hemodynamic Responses During Purdue Pegboard Testing

**FIGURE 6:**
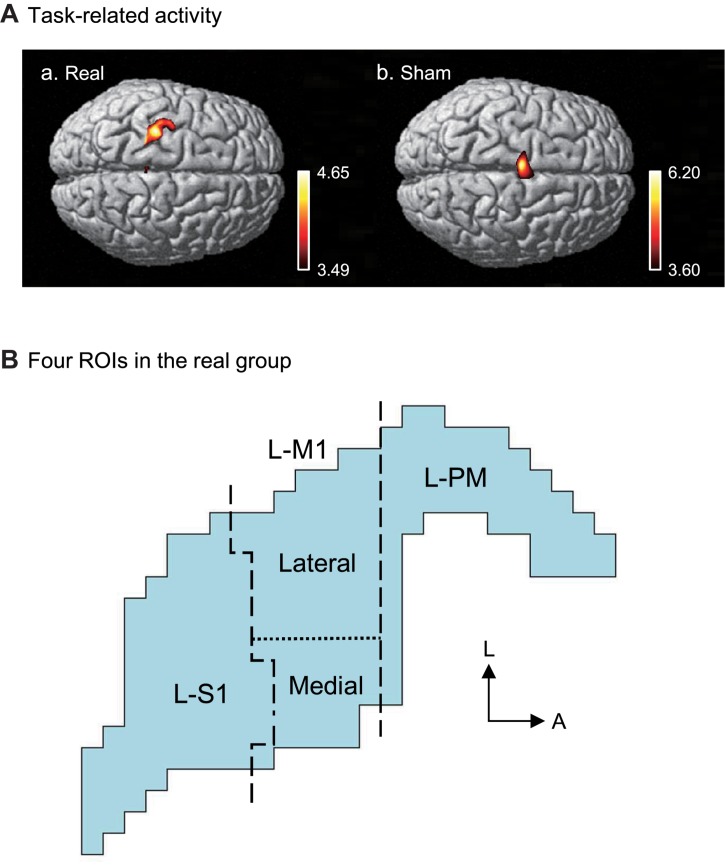
Task-related cortical activation during the Purdue Pegboard test in the post-assessment. **(A)** NIRS-SPM T-statistic maps in the real **(a)** and sham **(b)** groups. Task-related cortical activation was observed in the somatosensory motor-related areas in the real group **(a)**, and the supplementary motor area (SMA) in the sham group. **(B)** Schematic illustration of the task-related cortical activation in the real group. The task-related activated areas were divided into 4 ROIs: left premotor area (L-PM), lateral left primary motor area (L-M1) (lateral L-M1, hand motor area), medial L-M1, and the left primary somatosensory area (L-S1). L, lateral; A, anterior.

Figure 6 shows the contrast image maps during Purdue Pegboard testing in the post-assessment resulting from the group analysis based on the GLM with NIRS-SPM. In the real group, task-related cortical activation was observed in the somatosensory motor-related areas: L-PM, L-M1, and L-S1 (Figure 6Aa). The L-M1 was further divided into the hand area (lateral L-M1) and the remaining L-M1 (medial L-M1) (see section Materials and Methods). A schematic illustration of the activated areas is shown in Figure 6B. The averaged MNI coordinates [(X, Y, Z) mm] of each ROI were as follows; L-PM, [Averaged MNI coordinate; (−36, −18, 68) mm]; hand area in L-M1 (lateral L-M1), [(−34, −25, 72) mm]; the remaining L-M1 (medial L-M1), [(−27, −25, 75) mm]; and L-S1, [(−29, −30, 75) mm].

In the sham group, task-related activity was observed in the SMA (Figure 6Ab). The averaged coordinates of the SMA were −4, 10, and 74 (X, Y, Z) mm.

### Relationships Among Motor Imagery Training, Somatosensory-Motor Cortical Activity, and Performance Gain

**FIGURE 7:**
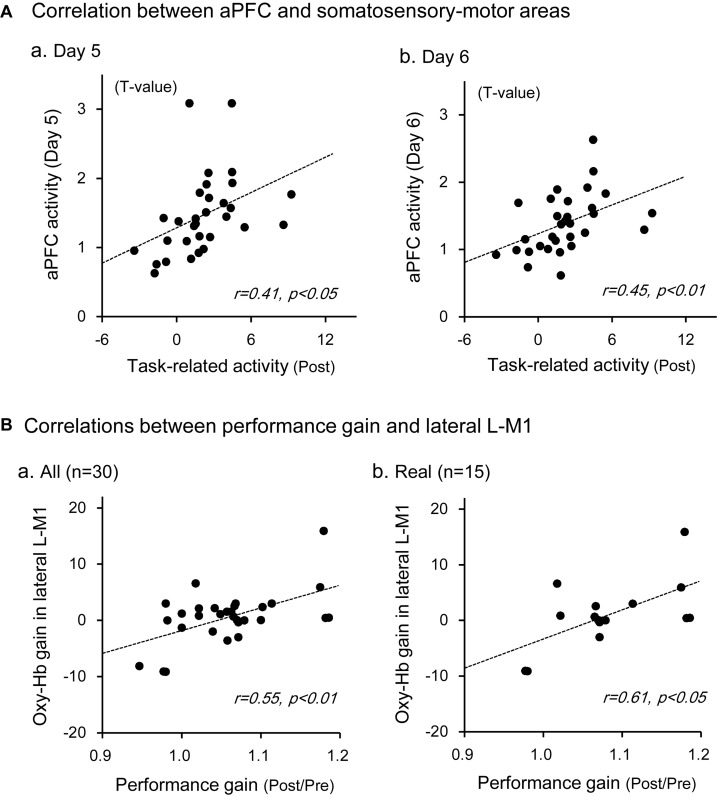
Relationships between aPFC activity during motor imagery training and activity in the somatosensory motor-related areas during the Purdue Pegboard test **(A)**, and those between Oxy-Hb gain in the lateral L-M1 and performance gain in the Purdue Pegboard test **(B)**. **(A)** There were significant positive correlations between the activity in the somatosensory motor-related areas and that in the aPFC on day 5 **(a)** and day 6 **(b)**. **(B)** There were significant positive correlations between Oxy-Hb gain in the lateral L-M1 and hand performance gain in the Purdue Pegboard test when data in the whole subjects **(a)** and subjects in the real group **(b)** were analyzed. The data in each circle indicate data from each subject.

The above data in Figure 5B indicated that Oxy-Hb gain in the aPFC during motor imagery training was significantly and positively correlated with performance gain. We hypothesized that motor imagery training gradually increased activity in the somatosensory motor-related areas through the aPFC, which in turn increased performance gain in the Purdue Pegboard test. First, we analyzed the relationships between aPFC activity during motor imagery training and activity in the somatosensory motor-related areas during Purdue Pegboard testing in the post-assessment (Figure 7A). Statistical analyses by a simple regression analysis indicated that task-related activation in the somatosensory motor-related areas (L-PM, L-M1, and L-S1) during Purdue Pegboard testing in the post-assessment significantly and positively correlated with aPFC activity on day 5 [day 5; *r* = 0.41, *F*(1,29) = 5.765, *P* < 0.05], and day 6 [day 6; *r* = 0.45, *F*(1,29) = 7.284, *P* < 0.05] in the motor imagery training. However, there were no such correlations on day 1, 2, 3, and 4 (data not shown).

Second, we then analyzed the relationships between Oxy-Hb gain in the somatosensory motor-related areas in the Purdue Pegboard test (i.e., improvement of task-related activation) and performance gain in the Purdue Pegboard test (i.e., improvement of hand dexterity). There were no significant relationships between Oxy-Hb gain in the entire activated somatosensory motor-related areas and performance gain [*r* = 0.35, *F*(1,29) = 4.068, *P* > 0.05]. However, there was a significant positive correlation between Oxy-Hb gain in the lateral L-M1 (hand motor area) and performance gain (Figure 7B). In the whole subject analysis (Figure 7Ba), data from one sample were removed as outliers by the residual analysis (standardized residual = −5.001), and there was a significant positive correlation between Oxy-Hb gain in the lateral L-M1 and performance gain [*r* = 0.55, *F*(1,28) = 12.201, *P* < 0.01]. When the data were confined to the real group (Figure 7Bb), data from one sample were also removed as outliers (standardized residual = −3.481), and there was also a significant positive correlation between Oxy-Hb gain in the lateral L-M1 and performance gain [*r* = 0.61, *F*(1,13) = 7.786, *P* < 0.05].

In contrast, no significant correlation was observed between Oxy-Hb gain in the SMA and performance gain in the sham group [*r* = 0.39, *F*(1,12) = 2.183, *P* > 0.05], where data from one sample were removed as outliers (standardized residual = −3.468), nor significant correlation in the whole subject analysis [*r* = 0.12, *F*(1,28) = 0.429, *P* > 0.05], where one sample data were also removed as an outlier (standardized residual = −5.200).

## Discussion

### Effects of Neurofeedback Training Targeting the aPFC

The neurofeedback training in healthy adult subjects significantly increased cerebral hemodynamic activity in the aPFC in the real group when compared with the sham group. These findings indicated that the subjects could volitionally control (self-regulate) aPFC hemodynamic activity. Previous studies also reported that subjects could self-regulate activity in specific brain areas including the PM, SMA, and aPFC through neurofeedback-guided motor imagery training (Mihara et al., 2012, 2013; Kinoshita et al., 2016; Subramanian et al., 2016). The present study further indicated that performance gain was significantly increased in the real group than the sham group after motor imagery training, and that motor imagery training progress (i.e., Oxy-Hb gain in the aPFC) correlated with performance improvement (i.e., performance gain) in the Purdue Pegboard test. These findings suggest that the activation of the aPFC is associated with improvement in hand motor functions. Consistent with this idea, our previous studies reported that Oxy-Hb gain in the aPFC positively correlated with performance gain during repeated training in a task similar to the Purdue Pegboard test, and that anodal stimulation of the aPFC by transcranial direct current stimulation (tDCS) increased performance in a motor rehabilitation task similar to the Purdue Pegboard test in healthy adults as well as in patients with Parkinson’s disease (Ishikuro et al., 2014, 2018). Furthermore, activity in the aPFC was reported to correlate with shoulder function after surgery due to shoulder dislocation (Zanchi et al., 2017). These findings suggest that the aPFC is an important target for neurofeedback training in motor rehabilitation.

### Effects of Neurofeedback Training on the Somatosensory Motor-Related Areas

In the present study, the SPM map in the group analysis indicated that hemodynamic activity in the somatosensory motor-related areas (L-PM, lateral L-M1, medial L-M1, and L-S1) increased during Purdue Pegboard testing after neurofeedback training in the real group with hand performance improvement. The results suggest that the left somatosensory motor-related areas are essential for motor skill learning using the right hand. Consistent with the present results, recent studies suggest that the motor cortex (L-M1), which plays a prominent role in movement control, is also important in motor skill learning (Papale and Hooks, 2018), and that the somatosensory cortex (L-S1) is also involved in motor control through its direct projections to the motor cortex (Matyas et al., 2010). The premotor cortex (L-PM) is also implicated in motor learning in healthy subjects as well as patients (Mihara et al., 2012, 2013; Hardwick et al., 2013). Transcranial direct current stimulation of the premotor cortex increased hand dexterity (Pavlova et al., 2014), and increased excitability of the ipsilateral M1 area (Boros et al., 2008), suggesting that L-PM effects on performance gain might be mediated through its effects on the L-M1. Furthermore, hemodynamic activity in the aPFC was positively correlated with that in the L-PM during motor learning in a similar motor task (Ishikuro et al., 2014). These finding suggest that the aPFC might affect motor learning through the L-PM and L-M1.

Interestingly, hemodynamic activity in the SMA increased in the sham group during Purdue Pegboard testing in the post-assessment. The SMA has been implicated in learning a new association between stimuli and motor responses, and in cognitive control to inhibit a response plan (Nachev et al., 2008). Furthermore, a recent intracranial recording study suggests that the SMA functions as an action-monitoring system to emit alarm signals for incorrect responses or errors (Bonini et al., 2014). In the sham group, the subjects received random feedback signals regardless of their own aPFC activation during motor imagery of the Purdue Pegboard test. This indicates that the correct aPFC activation that led to execution of the Purdue Pegboard test was not facilitated in the sham group, further suggesting that irrelevant aPFC activation might develop irrelevant synaptic activation in the somatosensory motor-related area in a way different from that used in the Purdue Pegboard test. Therefore, in the post-assessment of the Purdue Pegboard test, the subjects in the sham group might have to correct wrong synaptic activity formed by sham motor imagery training. Thus, the SMA activity in the sham group might increase to detect wrong synaptic activity in the somatosensory motor-related areas, to inhibit wrong responses, and to learn correct (new) association between incoming visual inputs and motor responses.

### Neural Mechanisms of Performance Improvement

Hemodynamic activity in the somatosensory motor-related areas increased in the Purdue Pegboard test after neurofeedback training in the real group. This activity increase correlated with aPFC activity on days 5 and 6 during motor imagery training. Furthermore, Oxy-Hb gain in the lateral L-M1 (hand motor area) positively correlated with hand performance gain in the Purdue Pegboard test. It has been proposed that there are two stages of motor skill learning; initial fast learning (e.g., within a single session of training) and late slow learning (e.g., repeated training over a month to increase accuracy and speed) (Dayan and Cohen, 2011). In an initial fast learning, BOLD signals in the M1 decrease along with progression of learning, while BOLD signals in the M1 gradually increase along with learning in a late slow learning (see a review by Dayan and Cohen, 2011). The present results indicating significant increases in Oxy-Hb in the lateral L-M1 after repeated motor imagery training for 6 days, which were positively associated with task performance, suggest that neural mechanisms for late slow learning may be involved in the present neurofeedback training.

Our previous results indicated that response latencies in the aPFC were faster than in the somatosensory motor-related areas, and that hemodynamic activity in the aPFC correlated with that in the somatosensory motor-related areas during motor learning in a similar motor rehabilitation task (Ishikuro et al., 2014). Non-invasive studies reported indirect projections from the aPFC to the somatosensory motor-related areas (Hasan et al., 2013; Liu et al., 2013). Human neuropsychological studies suggest that the activity of the anterior part of the PFC, including the aPFC, was increased when subjects learned new motor task(s) (Jenkins et al., 1994; Floyer-Lea and Matthews, 2004), and lesions to these PFC areas delayed motor learning (de Guise et al., 1999; Richer et al., 1999). These findings suggest that the aPFC might shape synaptic activity in the somatosensory motor-related areas to improve hand dexterity during neurofeedback motor imagery training. Induction of such synaptic plasticity during feedback training might be mediated through long-term potentiation (LTP)-like and long-term depression (LTD)-like mechanisms as well as through dopaminergic activity (Sitaram et al., 2016).

Consistent with this idea, previous human studies suggest that the motor learning process during the repetition of a motor task involves synaptic plasticity in the M1 area, including LTP- and LTD-like mechanisms (Rioult-Pedotti et al., 1998; Muellbacher et al., 2002; Jung and Ziemann, 2009). The dorsolateral PFC, which receives projections from the aPFC (Liu et al., 2013), could facilitate excitability of the ipsilateral M1 area (Hasan et al., 2013), consistent with LTP induction. Second, dopaminergic neurons receive direct and/or indirect glutamatergic projections from the PFC (Kalivas, 1993; Carr and Sesack, 2000; Omelchenko and Sesack, 2007; Han et al., 2017), and dopaminergic neuronal activity correlates with that of PFC neurons (Gao et al., 2007; Zhang et al., 2012), suggesting that aPFC activity might induce dopamine release in the somatosensory motor-related areas. Furthermore, dopamine facilitates LTP induction as well as motor skill learning (Li et al., 2003; Molina-Luna et al., 2009; Hosp et al., 2011). Taken together, the aPFC might improve performance gain partly through these two mechanisms.

### Limitations

There are several limitations in the present study. First, although performance in the Purdue Pegboard test was improved by motor imagery training to increase aPFC activity, motor imagery training itself could improve performance in the Purdue Pegboard test regardless of aPFC activity. Therefore, the sham group was introduced to control motor imagery training to exclude such possibility. Furthermore, the subjects were randomly assigned to the two groups, and there was no significant difference in motor imagery ability at least after the pre-assessment of the Purdue Pegboard test. However, sense of agency (“feeling of being in control”: Jeunet et al., 2016) could affect motor imagery performance. Although the subjects were blinded to subject grouping, they could identify subject grouping through their sense of agency during the motor imagery training. Identification of own group could affect degree of engagement in the motor imagery training. Thus, sense of agency could affect performance gain in the Purdue Pegboard test regardless of aPFC activity. However, the present results indicated that activity in the aPFC was significantly associated with activity in the somatosensory motor-related areas and performance in the Purdue Pegboard test. These results indicated that aPFC activity during the motor imagery training is one of the important factors to improve performance in the Purdue Pegboard test.

Second, we used young healthy subjects in the present study. However, patients with stroke as well as chronic neurological disorders such as Parkinson’s disease and Alzheimer’s disease are expected to undergo the neurofeedback training, and would be older than the present subjects. A previous study reported that aPFC stimulation by tDCS ameliorated hand dexterity in elder patients with Parkinson’s disease (Ishikuro et al., 2018), suggesting that the neurofeedback training to increase aPFC activity might be effective in elder patients. Third, we analyzed relationships between dexterity and hemodynamic activity in the left somatosensory motor-related area, since only this area was activated during the Purdue Pegboard test in the post-assessment session. However, activity in the ventral parts of the brain such the cerebellum and basal ganglia, which are also implicated in motor learning (Atallah et al., 2007; Spampinato and Celnik, 2017), were not investigated due to methodological limitation of NIRS. Fourth, although hemodynamic activity changes based on neurovascular coupling (see section Introduction), it cannot detect whether these changes are associated with neurophysiological facilitation nor inhibition. Fifth, we observed the motor behaviors during the motor imagery training by videos instead of EMG recording since it is difficult to record EMGs from all of the many muscles involved in stretching the arm and picking up a peg. However, video inspection could miss muscle activity without overt movements during the motor imagery training. Finally, sense of agency, which is an important factor for effectiveness of intervention with neurofeedback training (Braun et al., 2018), was not evaluated in the present study. However, sense of agency is reported to be positively associated with performance in neurofeedback training (Jeunet et al., 2016), and hemodynamic activity in the aPFC during the motor imagery training was larger in the real than sham groups, suggesting that sense of agency during the training might be higher in the real than sham groups. Further studies were required to evaluate usefulness of this neurofeedback training in patients with motor disabilities.

## Conclusion

In the present study, the healthy adult subjects were trained to increase aPFC activity by using motor imagery of the Purdue Pegboard test under real-time neurofeedback from the aPFC: the real group subjects received real feedback signals from the aPFC, whereas the sham group subjects received random signals. The motor imagery training significantly increased hemodynamic activity in the aPFC in subjects in the real group when compared with subjects in the sham group. After the training, group analysis of hemodynamic activity during Purdue Pegboard testing indicated that the somatosensory motor-related areas (L-PM, lateral L-M1, medial L-M1, and L-S1) were activated in the real group with hand performance improvement, while hemodynamic activity in the SMA was increased in the sham group. Furthermore, the hemodynamic activity in the somatosensory motor-related areas during the Purdue Pegboard test after the training correlated with the activity in the aPFC on the last two days during motor imagery training. In addition, Oxy-Hb gain in the lateral L-M1 positively correlated with hand performance gain in the Purdue Pegboard test. Motor skill learning requiring fine motor functions has been attributed to changes in neural circuits in the sensorimotor cortex (Hatakenaka et al., 2007; Papale and Hooks, 2018). The present results suggest that neurofeedback training from the aPFC might induce synaptic plasticity in the sensorimotor cortex. These findings further suggest that motor imagery training using neurofeedback from the aPFC can be applied to patients with stroke or chronic neurological disorders.

## Data Availability Statement

The data that support the findings in this study are available from the corresponding author HSN, upon reasonable request.

## Ethics Statement

The studies involving human participants were reviewed and approved by the Ethical Committee of Human Experiments at the University of Toyama. The participants provided their written informed consent to participate in this study.

## Author Contributions

HSN, SU, and MM designed the experiment. YO performed the experiment. YO and HisN analyzed the data and wrote the manuscript. YO, HSN, KT, HRN, SU, JM, YT, MM, and TO revised the manuscript. All authors discussed the results, and approved the final manuscript.

## Conflict of Interest

The authors declare that the research was conducted in the absence of any commercial or financial relationships that could be construed as a potential conflict of interest.

## Footnotes

1 http://www.gpower.hhu.de/

2 http://www.MRIcro.com

3 https://www.nitrc.org/projects/nirs_spm/
