# Supplementary material for: Motor Imagery Training With Neurofeedback From the Frontal Pole Facilitated Sensorimotor Cortical Activity and Improved Hand Dexterity
Source: Front Neurosci. 2020 Jan 29;14:34. doi: 10.3389/fnins.2020.00034 (PMC7025527; doi:10.3389/fnins.2020.00034)
Supplement: Supplementary file 1 [file Data_Sheet_1.PDF]

Supplementary Material

Supplementary Figure 1.

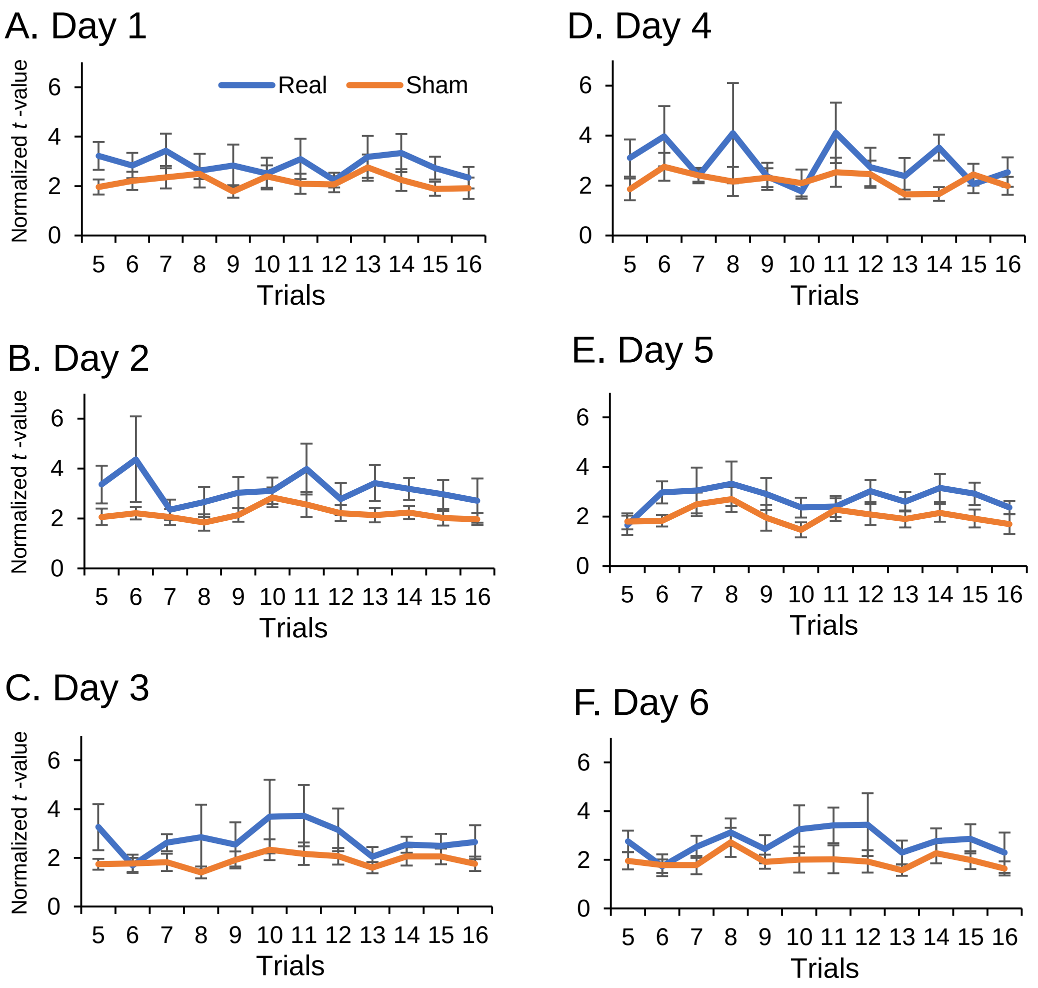

Supplementary Figure 1. Changes in mean *t*-values of the aPFC activity during neurofeedback training over 6 days.
A-F: Changes in normalized *t*-values of aPFC activity from day 1 (A) to day 6 (F). To observe trends of changes in aPFC activity (averaged *t*-values across Ch 1-5), data in each day were normalized by the minimum *t*-values in the trials 1-3 on Day 1 in each subject. The data in the initial four trials were not analyzed (see Methods).
